# Endoplasmic Reticulum Stress and Intestinal Inflammation: A Perilous Union

**DOI:** 10.3389/fimmu.2020.543022

**Published:** 2020-11-25

**Authors:** Sanchez Preethi Eugene, Vadde Sudhakar Reddy, Jamma Trinath

**Affiliations:** ^1^ Department of Biological Sciences, Birla Institute of Technology and Science-Pilani, Hyderabad Campus, Hyderabad, India; ^2^ Biochemistry Division, National Institute of Nutrition, Hyderabad, India

**Keywords:** endoplasmic reticulum stress, unfolded protein response, apoptosis, inflammation, intestinal epithelial cells

## Abstract

The intestinal tract encompasses the largest mucosal surface fortified with a fine layer of intestinal epithelial cells along with highly sophisticated network of the lamina propria immune cells that are indispensable to sustain gut homeostasis. However, it can be challenging to uphold homeostasis when these cells in the intestine are perpetually exposed to insults of both endogenous and exogenous origin. The complex networking and dynamic microenvironment in the intestine demand highly functional cells ultimately burdening the endoplasmic reticulum (ER) leading to ER stress. Unresolved ER stress is one of the primary contributors to the pathogenesis of inflammatory bowel diseases (IBD). Studies also suggest that ER stress can be the primary cause of inflammation and/or the consequence of inflammation. Therefore, understanding the patterns of expression of ER stress regulators and deciphering the intricate interplay between ER stress and inflammatory pathways in intestinal epithelial cells in association with lamina propria immune cells contribute toward the development of novel therapies to tackle IBD. This review provides imperative insights into the molecular markers involved in the pathogenesis of IBD by potentiating ER stress and inflammation and briefly describes the potential pharmacological intervention strategies to mitigate ER stress and IBD. In addition, genetic mutations in the biomarkers contributing to abnormalities in the ER stress signaling pathways further emphasizes the relevance of biomarkers in potential treatment for IBD.

## Introduction

The intestine houses a plethora of innocuous microbes that establish a symbiotic relationship in the host. Additionally, constant exposure to the external factors makes it susceptible to invasion by exogenous pathogens ensuing persistent immune response in the gut. Therefore, the lamina propria immune cells must be functionally fine-tuned to differentiate and exhibit tolerance toward commensals and immunity to pathogens. At this juncture, the intestinal epithelial cells (IECs) lining the gut play two major roles: segregation and mediation, conserve gut homeostasis (1); avoid unwarranted immune responses to gut microbes utilizing highly specialized cell types (Paneth cells, goblet cells, enteroendocrine cells, and absorptive epithelial cells) (2–7). Perturbations in the functions of these IECs cause microbial dysbiosis, infiltration and hyperactivation of immune cells in the lamina propria contributing to IBD. IBD is multifactorial whose pathophysiology is unclear and disrupts several aspects such as the physiology, microbiology, immunology, and genetics of the host mimicking a chaotic battlefield. Simply put, the impairment of one aspect causes the annihilation of the other. One such widely reported contributing factor for IBD is ER stress as described below.

## ER Stress and UPR

The ER is the primary site for facilitating the appropriate folding of proteins and dispatches them to their respective functional destinations in the IEC with secretory function (2). However, the proteins that shuttle through the ER may aggregate, triggering a highly conserved unfolded protein response (UPR) and establish ER homeostasis in three possible ways (8). A. Transcriptional induction:- increases the protein folding capacity by transcribing chaperones that aid in proper folding; B. Translational attenuation:- reduces protein load in the ER by arresting translational machinery, degrading mRNAs; C. ER-associated degradation:- the unfolded proteins are marked for proteasomal degradation. However, if ER stress persists, the effort to establish homeostasis can be futile triggering apoptosis in IEC (9).

## UPR Signal Transducers

UPR pathways function with unique mechanisms of signal transduction operating in parallel utilizing IRE1α, PERK, and ATF6 (9). In their inactive state, these stress sensors are bound to BiP toward the intraluminal domain. Under ER stress, the BiP dissociates, activating IRE1α, PERK, and ATF6 signaling cascades to salvage the distressed cell.

**Figure 1:**
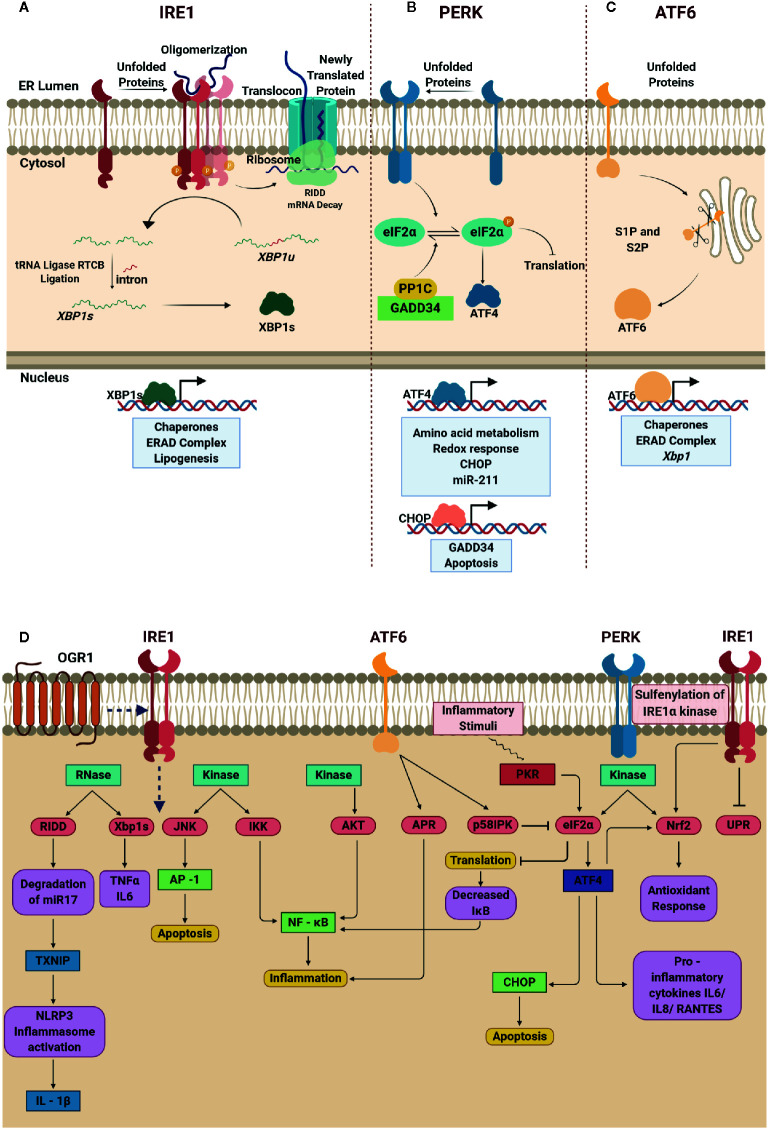
Schematic depiction of unfolded protein response (UPR) signaling cascade, and the interplay between endoplasmic reticulum (ER) stress response and inflammation. **(A)** Oligomerization of IRE1α in the presence of unfolded proteins promotes the endoribonuclease activity of IRE1α and unconventional splicing of XBP1 generating functional XBP1 that regulates gene expression. IRE1α promotes RIDD-dependent mRNA decay and reduces protein overload in the ER lumen. **(B)** Activated PERK drives phosphorylation of eIF2α resulting in translational block. At this juncture, selective IRES dependent translation of ATF4 is promoted to induce chaperones and mitigate oxidative stress as well as apoptosis. **(C)** Dissociation of BiP from ATF6 leads to translocation of ATF6 from the ER membrane to Golgi promoting its cleavage by S1P and S2P generating functional ATF6 that regulates UPR genes. **(D)** In IRE1α pathway, the RNase domain is involved in Xbp1 splicing and RIDD mechanism upregulates the expression of pro-inflammatory cytokines TNFα, IL-6 and IL-1β; the kinase domain activates JNK and IKK signaling pathway that results in apoptosis and inflammation respectively. Additionally, activation of proton-sensing OGR1 is responsible for ER-stress mediated response *via* IRE1α-JNK-XBP1s axis. The kinase activity of ATF6 leads to phosphorylation of AKT ensuing inflammation *via* NF-κB signaling. The cleaved p50ATF6α acts as a transcription factor and upregulates the expression of APR genes and ER co-chaperone p58IPK that in turn blocks the phosphorylation of eIF2α. PERK is one of the kinases that phosphorylate eIF2α at Ser 51, which enables selective translation of ATF4. ATF4 drives the expression of CHOP and pro-inflammatory cytokines such as IL-6, IL-8, and RANTES. Induced expression of CHOP abrogates pro-survival signaling leading to cell death. Notably, the translational block decreases further translation of IκB ensuing inflammation due to increase in NF-κB. Nrf2, another notable target phosphorylated by PERK that is known to manifest antioxidant response. Created with BioRender.com.

**IRE1α** is the most evolutionarily conserved transmembrane kinase with endoribonuclease activity (10). The active IRE1α cleaves the 26-nucleotide intron from *XBP1* forming functional *XBP1s* (11), which then enters the nucleus, and regulate UPR-related genes. Interestingly, the XBP1u is degraded rapidly after translation; however, during prolonged stress, XBP1u is reported to accumulate and complex with XBP1s, to promote ubiquitin-mediated degradation of XBP1s in HeLa cells (12). Therefore, a balanced level of XBP1u and XBP1s partly dictates the functional role of IRE1α. Another important regulatory mechanism executed by IRE1α is through IRE1-dependent mRNA decay (RIDD). IRE1α cleaves the transcripts that enter ER through the translocon and prevents accumulation of unfolded proteins in the ER (13). Nevertheless, RIDD can also be deleterious if mRNAs that translate for pro-survival proteins are degraded suggesting that a selective degradation of mRNA is favored. Of note, in the recent past, the ability of RIDD pathway to degrade microRNAs responsible for inhibiting the translation of CASP2 in mouse embryonic fibroblasts (MEF) have also been identified signifying that fine-tuning the availability of non-coding RNAs also contribute to the overall outcome of UPR (14) (**Figure 1A**).

**PERK** is a type 1 transmembrane serine/threonine kinase, when bound to BiP, remains inactive (10). Dissociation of BiP enables dimerization of PERK and promotes its kinase activity and phosphorylates eIF2α causing a translational block to manage ER stress. Another pivotal step that occurs at this stage is the selective internal ribosomal entry site mediated translation of ATF4 amidst the inhibitory phosphorylation of eIF2α (15). Nuclear translocation of ATF4 promotes GADD34, CHOP and miR-211 expression (16, 17) to mediate UPR in mouse embryonic fibroblasts. Upon resolution of ER stress, GADD34 complexes with PP1C and dephosphorylates eIF2α to restore protein translation. Interestingly, PERK-induced miR-211 abrogates the expression of CHOP/GADD34 suggesting the pro-survival role of miR211 (17). However, if the ER stress remains unresolved, CHOP activates the terminal UPR to induce apoptosis in IEC (18, 19) (**Figure 1B**).

**ATF6** is also a transmembrane kinase, with a basic leucine zipper (bZIP) domain, unlike IRE1α and PERK. Once the BiP dislodges from ATF6, it is trafficked from the ER to Golgi. S1P and S2P cleaves ATF6 releasing the N-terminal cytosolic domain of ATF6 (N) that translocate to the nucleus and promotes the transcription of chaperones, ERAD complex and XBP1 to mitigate ER stress (**Figure 1C**).

In addition to the three primary signal transducers, a few ER stress transducers belonging to the OASIS family are identified recently (20). These stress sensors share a region of high sequence similarity with ATF6. One such example is CREBH, which is also trafficked from ER to Golgi and proteolyzed by S1P and S2P (21). Studies indicate that ATF6 and CREBH regulate inflammatory gene expression during the early phase of infection or injury.

## UPR and Inflammation

Recent studies have extended a clear understanding of the relationship between UPR signaling and inflammation (**Figure 1D**). Activated IRE1α, in addition to its role as bifunctional enzyme, interacts with TRAF2 to activate JNK and NF-κB (22, 23) regulating inflammatory gene expression. Studies also indicate that XBP1s induces TNFα and IL-6 (24, 25) which in turn activate NF-κB (23, 26); thus amplifying the inflammatory responses. Interestingly, degradation of miR-17, a microRNA that represses the expression of thioredoxin-interacting protein (TXNIP) by IRE1α results in stabilization of TXNIP and expression of IL-1β (27, 28). At the molecular level, identification of IRE1α-TXNIP axis to activate NLRP3 inflammasome, IL-1β expression and programmed cell death (27) through miR-17 degradation hint that ER stress regulates inflammation. Furthermore, activation of IRE1α-GSK3β axis induces the expression of IL-1β and regulates the expression of TNFα (29). It is also a known fact that GSK-3β requires priming kinase to phosphorylate the substrate first for recognition and phosphorylates the phosphorylated substrate at a different site (30). Therefore, it is conjectured that the phosphorylated kinase domain of IRE1α acts as the priming kinase for GSK-3β to phosphorylate the riboendonuclease domain of IRE1α rendering it inactive leaving the hypothesis to be tested as a future prospect. Inhibition of global translation upon activation of PERK lowers the levels of IκBα ensuing massive activation of NF-κB (31). On the other hand, the selectively translated ATF4 binds to IL-6 promoter and regulates its expression (32). Of note, TLR4 signaling is also responsible for the induction of ATF4, independent of ER stressors, resulting in the transactivation of *Il6, Il18, and Rantes* in macrophages and monocytes (32, 33). Additionally, PERK also directly phosphorylates NRF2, regulating the antioxidant response by nullifying the ROS production during ER stress in fibroblasts (34). Recent investigations, however, direct toward an alternative NRF2 regulation in response to oxidative stress *via* PERK-eIF2α-ATF4 axis in human cells (NCI-H358) (35). Reversible sulfenylation of cysteine residue (C663) of IRE1α by ROS results in the attenuation of UPR and promotes activation of NRF2-mediated antioxidant response in human cells (36). Overall activation of PERK inhibits eukaryotic translation, prevents the accumulation of unfolded proteins that promote inflammatory gene expression, and regulates apoptosis through ATF4. Interestingly, similar to IRE1α and PERK, ATF6 also contributes to NF-κB signaling through transient phosphorylation of AKT, however, prolonged ER stress resulted in downregulation of AKT phosphorylation (37). The phospho-refractory nature of AKT after ATF6-mediated ER stress is further confirmed by subsequent TLR4 stimulation of ischemic Kupffer cells (38). However, the ability of ATF6 to regulate inflammatory gene expression remains unexplored to a large extent.

## ER Stress and UPR in Intestinal Inflammation

Continual exposure to a myriad of gut microflora, exogenous antigens, dietary metabolites, and toxins impede the functional ability of IECs. Although nature has bestowed with evolutionarily conserved and sophisticated mechanisms to overcome these impediments, disruption in any of these mechanisms can cause chronic inflammation in the gut. Accordingly, there are two ways to look at the cause for the collapse of these mechanisms: i) IECs are pushed to synthesize copious amounts of proteins, cytokines, and peptides; activating UPR. In this scenario, cells that are competent enough will survive and the rest will succumb to stress. ii) Genetic deficiency of the genes that are involved in UPR, autophagy, secretion, immune response and inflammation can have various impacts and confer a genetic predisposition to IBD owing to decreased protein folding capacity and heightened immune response. Mechanistic studies conducted on murine models deficient in these genes facilitated the understanding of phenotypic outcomes in correlation to IBD (**Table 1**).

**Table 1:** List of endoplasmic reticulum (ER) stress-related genes in inflammatory bowel diseases (IBD).

Gene	Function	Implications
*Ire1α*	*Xbp1* splicing, RIDD, activation of JNK and NF-κB signaling (8, 9)	*Xbp1* splicing, Enhanced CHOP – induced apoptosis (39)
*Xbp1*	Transcription factor – Chaperones, ERAD complex, Lipid biosynthesis (8, 9)	Ire1α hyperactivation, Amplified ER stress, Increased JNK phosphorylation, Heightened expression of pro – inflammatory genes (40)
*Ire1β*	Selective repression of ER – localized secretory proteins (41)	Aberrant accumulation of secretory proteins (42)
*Chop*	Transcription factor – Increases protein load in ER by dephosphorylation of eIF2α, Induction of apoptotic signalling (18, 43)	Decreased apoptosis (44)
eIF2α phosphorylation	Regulatory node in maintaining cellular homeostasis, Attenuation of global mRNA translation, Selective translation of ATF4 (9)	Defective expression of UPR genes, Defective recruitment of secretory protein coding mRNAs into the ER leading to compromised protein secretion (45)
*Atf6α*	Membrane – bound transcription factor – *Xbp1*, Chaperones, ERAD complex (8, 9)	Diminished expression of ER chaperones BiP and P58IPK, CHOP – induced apoptosis (46, 47)
*Atg16l1*	Regulates autophagy; autophagosome formation	Impaired granule exocytosis pathway in Paneth cells (48), increased ATF6α activity (49) and IL-22 induced TNF expression leading to necroptosis (50)

Of the three pathways, IRE1α-XBP1 axis of UPR has been extensively studied and known to play an essential role in regulating immunity and inflammation. Additional evidence from the studies suggests that *Xbp1* is linked to IBD. Cell-specific loss of *Xbp1* in intestinal epithelial cells (*Xbp1^ΔIEC^*) displayed amplified ER stress (40). Additionally, deep-sequencing studies have revealed rare variants/SNPs of the *Xbp1* gene that contributes to the susceptibility and severity of the inflammatory disorders in humans (40). Furthermore, *Xbp1* deletion resulted in hyperactivation of IRE1α and enhanced the susceptibility to experimentally induced inflammation in mice suggesting a pivotal role of IRE1α in intestinal inflammation. Studies demonstrated that IRE1α recruits TNFR1 during ER stress to activate TNF-independent JNK signaling and apoptosis (51). This was further supported by the fact that the deletion of *Tnfr1* in *Xbp1^ΔIEC^* mice failed to develop intestinal inflammation and the deletion of *Xbp1* in IECs resulted in the apoptotic loss of Paneth cells that maintain homeostasis (40). Of note, ER stress can also activate TNF-independent TNFR1-mediated necroptosis, a programmed RIPK1/RIPK3/MLKL-dependent necrosis, in L929 cells. Inhibition of JNK, however, resulted in the inhibition of both TNFR1-mediated apoptosis and necroptosis (52). On the other hand, the induction of CHOP in *Xbp1^ΔIEC^* upregulated the expression of induced NKG2D ligand, which activates natural killer cell-mediated cytotoxicity establishing the involvement of CHOP in innate immune responses (53). Moreover, genetic deletion of *IRE1α* led to impaired *XBP1* splicing and JNK-driven phosphorylation of eIF2α through PERK that promotes apoptotic cell death, suggesting a prominent pro-survival role of IRE1α as well. Furthermore, the study indicated compromised intestinal epithelial barrier integrity, lymphocyte infiltration and induced expression of TNFα, IL-1β and IL-6 leading to the development of spontaneous colitis in the mice (39). Altogether, these studies implicate a homeostatic role of IRE1α in mucosal immunity.

At later stages of ER stress, the PERK-ATF4 axis of UPR is predominantly active and induces CHOP. Whole-body deletion of *Chop* in mice suppressed the induction of Mac-1, Ero-1α, and caspase-11 with reduced intestinal epithelial cell apoptosis (43, 44). Another important component of the PERK pathway is phosphorylated eIF2α and its role has been studied using IEC-specific non-phosphorylatable S51A mutant *AA^ΔIEC^* mice (45). The translocation machinery to recruit mRNA into ER has been found to be defective in *AA^ΔIEC^* mice leading to defective antimicrobial peptides, cryptidin, and lysozyme resulting in the breach of epithelial integrity by commensals and hyperactivation of immune cells. Another notable feature is that a family of protein kinases such as PKR, GCN2, and HRI other than PERK phosphorylate eIF2α and regulate ER stress. Although these protein kinases are activated by different stimuli including infections and inflammatory cytokines, they culminate into phosphorylation of eIF2α at Ser51 emphasizing the importance of eIF2α in maintaining IEC homeostasis (54).

The functional role of *Atf6α* has been experimented using *Atf6^-/-^* mice wherein deletion of *Atf6α* led to reduced expression of ER chaperones BiP and p58IPK and showed signs of apoptosis (46). *p58IPK^-/-^* mice showed amplified ER stress and were more susceptible to DSS-induced colitis (55). Deletion of both *Atf6α* and *p58IPK* resulted in embryonic lethality, suggesting either ATF6α or p58IPK is required to oversee protein-folding defects (56). It is important to note that p58IPK is an ER co-chaperone that negatively regulates eIF2α, which in turn down regulates ATF4 and CHOP. As indicated previously, owing to the structural and sequential similarity, *Oasis^-/-^* mice developed characteristics of IBD as observed in *Atf6^-/-^* mice impacting goblet cell maturation (57, 58). Studies in a similar mouse model elucidated the role of S1P-ATF6 axis in IBD and concluded that missense mutation in *Mbtps1* impaired ATF6 arm of UPR (59). Deficiency of *Atg16l1* and *Xbp1* genes shows increased activity of ATF6α. Interestingly, inhibition of the ATF6α co-activators, CSNK2B and ASCL1 reduced the activity of ATF6α and attenuated CXCL1 and TNFα expression (49). Furthermore, *Atg16l1^ΔIEC^* is responsible for IL-22-mediated activation of IFN1-TNF axis and ER stress response (50). Increased levels of TNF potentiate IL-22-induced necroptotic epithelial cell death, contradicting the previously reported protective role of IL-22. In addition, targeting IL-22 in TRUC mice model alleviated ER stress response and colitis (60). Hence, the paradoxical nature of IL-22 challenges its prospect as a treatment for IBD.

In addition to the aforementioned genes, there are other IBD risk genes such as *AGR2* and *Ormdl3*. AGR2 belongs to the PDI family; expressed strongly in tissues that secretes mucus and expressed abundantly in the inflamed mucosa of UC patients (61, 62). *Agr2^-/-^* knockout mice developed spontaneous granulomatous ileocolitis (63). In addition to its intracellular role, modifications in the KTEL motif of AGR2 implicate its role in protein secretion (64). To emphasize an interesting hypothesis that inflammation can induce ER stress, the *Il10^-/-^* mice model was studied and found that IL-10 mitigates intestinal inflammation during ER stress (65). A recent study, however, demonstrated ER stress in LPS-stimulated macrophages, abrogated the immunosuppressive effects of IL-10 (66). Acidic milieu in IBD activates OGR1 receptor found in abundance lining the mucosal region. Further evidence indicates the role of TNF (67) in the expression of OGR1 that in turn mediates ER stress and exacerbates inflammation *via* IRE1-JNK-XBP1s axis and blocks autophagy (68). Interestingly, deletion of OGR1 in *Il10^-/-^* female mice protected from the development of spontaneous colitis (67).

## Microbiota, ER Stress and Inflammation

As mentioned earlier, activation of UPR cascade as a consequence of ER stress potentiates inflammation and IBD (**Figure 1D**). Of note, evidences report impaired UPR signaling cascade in IBD (69, 70). In the recent past, the impact of diet, nutrients and gut microbiota have been implicated in ER stress and IBD (71). Prevalence of *Fusobacterium* activates UPR and promotes inflammation in UC patients (72). Probiotic bacteria such as *Lactobacillus paracasei* ameliorated intestinal inflammation through ER stress-UPR pathway (73). On the contrary, *Lactobacillus acidophilus* mitigated intestinal inflammation by suppressing NF-κB and thereby inhibiting ER stress (74). Adding to this, methyl deficient diet aggravates DSS-induced colitis by promoting ER stress (75). Interestingly, HFD driven ER stress has been found to be harmful as well as beneficial in ERs stress-mediated inflammation-driven osteoarthritis and liver pathology respectively (76, 77). Likewise, three cancer mice models fed with low protein diet reportedly activated IRE1α/RIG pathway in tumor cells limiting tumor growth (78). DSS treatment disrupts ER homeostasis and membrane integrity (79). Dietary administration of *Lachnum* Polysaccharide (LEP) to DSS-induced colitis mitigated ER stress-mediated inflammation not only by precluding immune cell infiltration, but also improved epithelial barrier integrity by regulating tight junction (TJ) proteins, mucus layer protecting proteins, and antimicrobial peptides (80). Altogether, these results, suggest a pivotal role for dietary components, microbiota and ER stress in inflammation of the intestine. However, a detailed investigation still remains to understand the molecular association among these to cause IBD.

## Potential Therapeutics Targeting ER Stress in IBD

As discussed earlier, dysfunctional ER stress and UPR is one of the contributing factors in the etiology of IBD (40, 81). Therefore, drugs targeting to alleviate ER stress appear as a convincing choice to treat IBD. Chemical chaperones such as TUDCA and 4-PBA augment protein folding and suppress ER stress in IECs *in vitro* (46, 82). Moreover, oral administration of TUDCA and 4-PBA reduced ER stress in *Il10^-/-^* mice and DSS-induced colitis in *P58IPK^-/-^* and *Atf6^-/-^* mice (46, 83). Recent studies conducted on NEC mouse models showed that TUDCA is capable of reducing the ER stress markers and apoptosis by inhibiting PERK-eIF2α *via* activation of the PI3K/Akt pathway (84). Salubrinal, a specific eIF2α phosphatase inhibitor, reduces tunicamycin-induced ER stress and TNFR1-independent necroptosis in hepatocytes by selectively preventing eIF2α dephosphorylation (85). The secondary bile acid UDCA protects the intestinal barrier by inducing epithelial cell migration at the site of injury (86) and ameliorates LPS-induced intestinal inflammation (87). In addition, studies conducted on DSS-induced colitis mice model demonstrated the ability of UDCA and LCA to mitigate colonic inflammation by inhibition of epithelial apoptosis (88). Amino acids such as L-glutamine and L-arginine have been reported to regulate proliferation and differentiation of IECs suggesting the role for dietary supplements to regulate ER stress (89, 90). Moreover, it has been proved that L-glutamine and glycine supplementation can salvage IECs from ER stress and apoptosis by improving the intestinal epithelial barrier function (91) upregulating tight junction proteins (92). Plant-based active ingredient berberine (BBR) has long been known to alleviate ER stress response as an alternative to chemical compounds. Accordingly, a recent study asserted the ability of berberine to reduce inflammation and apoptosis in DSS-induced colitis mice model (93). Furthermore, evidences suggest that PERK, and IRE1α inhibitors may be extended to IBD pathogenesis. Accordingly, STF-083010, a small molecule inhibitor that specifically targets IRE1α has been proven to reduce ER stress-driven inflammation in atherosclerosis and diabetes (94, 95). Similarly, pharmacological inhibition of PERK by GSK2656157 and GSK2606414 ameliorate tumor growth and enhance neuroprotection (96–98) and PKR inhibitors such as imoxin and 2-aminopurine reduced ER stress in mouse beta TC-6 cell line (99). Activator of ATF6, Compound 147 has shown to reduce the risk of infarction and preserve cardiac function (100). Nevertheless, the efficacy and implications of these small molecules in ER stress-driven intestinal inflammation remains to be largely unexplored.

## Conclusion

The incidence rate of IBD, which has been once considered the disease of the developed nations, is alarmingly at a rise globally. Efforts have been placed to rationalize the root cause by postulating various hypothesis including hygiene (101) and cold chain hypothesis (102) culminating to a single root cause ‘microbial dysbiosis. There are numerous factors at play in disrupting the gut microbiome and integrity of the intestinal barrier (40). As a result, the compromised epithelial barrier allows breaching by microbes and exogenous antigens attracting the attention of the host’s immune system that tries to salvage but ends up damaging the host tissue due to inflammation. Chronic inflammation is one of the hallmark features of IBD, identical to a ‘wildfire’ that if uncontrolled causes collateral damage. There is also growing evidence that deregulated ER stress and UPR signaling pathways can instigate or magnify the inflammatory response in IBD (103–106). Therefore, restoring a robust ER stress and UPR mechanism could be a potential therapeutic target. Nevertheless, the lack of well-demarcated molecular pathways is downright challenging to develop targeted therapies to preclude overlapping adverse effects. However, the development of optimized therapeutics is possible if a profound understanding of the phenotype and pathogenesis of the disease can be established by delineating the cellular and molecular pathways.

## Author Contributions

SE and JT wrote the manuscript. VR and JT edited the manuscript into its final format. All authors contributed to the article and approved the submitted version.

## Funding

This work is supported by OPERA Award (BITS-Pilani, Hyderabad).

## Conflict of Interest

The authors declare that the research was conducted in the absence of any commercial or financial relationships that could be construed as a potential conflict of interest.

## Abbreviations

AGR2, Anterior Gradient 2; AKT, Protein Kinase B; APR, Acute Phase Response; ASCL1, Achaete-scute homolog 1; ATF4, Activating Transcription Factor; ATG16L1, Autophagy related 16 like 1; CASP2, Caspase 2; CHOP, C/EBP (CCAAT/Enhancer Binding Protein) Homologous Protein; CREBH, cAMP Responsive Element Binding Protein H; CRP, C – Reactive Protein; CSNK2B, Casein Kinase 2 Beta; DSS, Dextran Sulfate Sodium; eIF2α, Eukaryotic Initiation Factor 2α; ERAD, Endoplasmic Reticulum Associated Protein Degradation; Ero1α, Endoplasmic Reticulum Oxidoreductase 1α; GADD34, Growth Arrest and DNA Damage–Inducible Protein; GCN, General Control Nondepressible 2; HIK, Sensor Histidine Kinase/Response Regulator; IEC, Intestinal Epithelial Cells; IL–1β, Interleukin 1β; IL–6, Interleukin 6; JNK, c-Jun N- terminal Kinase; LCA, Lithocholic Acid; Mac-1, Macrophage-1 antigen; Mbtps1, Membrane Bound Transcription Factor Peptidase, Site 1; MLKL, Mixed Lineage Kinase domain-like Pseudo kinase; MUC2, Mucin 2; NEC, Necrotizing Enterocolitis; NF-κB, Nuclear Factor Kappa – Light Chain Enhancer of Activated B cells; NKG2D, Natural Killer Group 2D; NRF2, Nuclear Factor Erythroid 2–Related Factor; OASIS, Old Astrocyte Specifically Induced Substrate; OGR1, Ovarian cancer G-protein coupled receptor; ORMDL3, ORM1-like protein 3; 4– PBA, 4–Phenyl Butyrate; PERK, Protein Kinase R–Like ER Kinase; PI3K, Phosphatidylinositol 3–kinase; PKR, Protein Kinase R; PP1C, Protein Phosphatase 1C; RIDD, Regulated Ire1–Dependent mRNA Decay; RIPK1/3, Receptor-interacting serine/threonine protein kinase 1/3; S1P, Site 1 Protease; S2P, Site 2 Protease; SAP, Serum Amyloid Protein; SNP, Single Nucleotide Polymorphism; TNFα, Tumor Necrosis Factor α; TNFR1, Tumor Necrosis Factor Receptor 1; TRAF2, TNF Receptor–Associated Factor 2; TRUC, *T-bet^-/-^*/*RAG2^-/-^*/Ulcerative Colitis; TUDCA, Tauroursodeoxycholic Acid; UDCA, Ursodeoxycholic Acid; ULBP1, UL16 Binding Protein 1; XBP1s, X–box Binding Protein 1 spliced variant; XBP1u, X–box Binding Protein 1 unspliced variant.
